# Risk of Mood and Anxiety Disorders in the Postpartum Period Following Assisted Reproduction: A Retrospective Cohort Study

**DOI:** 10.1002/hsr2.72169

**Published:** 2026-04-02

**Authors:** Marisa R. Imbroane, Hanna Kim, Elliott G. Richards

**Affiliations:** ^1^ School of Medicine Cleveland Case Western Reserve University Cleveland Ohio USA; ^2^ Department of Reproductive Endocrinology and Infertility Cleveland Clinic Cleveland Ohio USA

**Keywords:** assisted reproductive technology, database, disparities, postpartum depression, postpartum mood disorders

## Abstract

**Background and Aims:**

To assess the risk of developing postpartum mood and anxiety disorders (PMAD) in patients who conceive via assisted reproductive technology (ART) versus naturally and to evaluate differences stratified by race and ethnicity.

**Methods:**

This retrospective cohort study was conducted using the TriNetX research network (> 100 million patients from 68 United States health care organizations). The ART group was defined using ICD‐10 code O09.81 (“supervision of pregnancy resulting from assisted reproductive technology”) and was compared against non‐ART pregnant, age, race, ethnicity, and history of mental health condition‐matched, non‐ART, controls, all of whom reached term gestation. The relative risk of developing PMAD within 12 months of delivery was investigated.

**Results:**

There were 43,103 patients in the ART group and 1,296,725 patients in the non‐ART group. Patients who conceived with ART were at a small but statistically increased risk for developing a depressive mood composite: postpartum depression, major depressive disorder, and depressive episode (5.9% vs 4.6%, RR 1.28 [95% CI (1.23,1.34)]), postpartum depression on its own (3.9% vs 3.4%, RR 1.13 (1.07,1.18)), and anxiety in the postpartum period (1.8% vs 1.2%, RR 1.56 [1.45,1.67]). These differences persisted when matching for both female infertility and obstetric complications. Within the ART group, Asian patients were significantly less likely than White patients to be diagnosed with all three outcomes.

**Conclusions:**

While patients who conceive via ART are at a slightly increased risk for PMAD, the difference is small and arguably not clinically significant. This may be related to the psychological burden and stress of an infertility diagnosis and infertility treatment. Mental health services should be offered to patients being seen in ART practice.

## Summary

- Assisted reproduction is associated with only a slightly increased risk for postpartum mood and anxiety disorders.
- Findings persist when matching for female infertility and obstetric complications.
- Asian patients were at a lower risk than their White counterparts for postpartum mood disorder development.

## 1 Introduction

Postpartum depression (PPD) is a mood disorder classified by a major depressive episode during the postpartum period, most commonly occurring within 4 to 8 weeks of delivery [1]. In prior studies, the prevalence of PPD has been estimated to be 8.6% in the United States with others estimating the prevalence of major depression alone at 1.0%–5.9% [2, 3]. PPD is regarded as a public health concern, disrupting the early mother‐infant relationship and being linked to adverse childhood outcomes like behavioral concerns, delayed development, and decreased self‐esteem [4, 5]. Other common psychiatric disorders affecting patients in the postpartum period include generalized anxiety disorder, as women in the postpartum period are at an increased risk for anxiety disorders compared to the general population [6].

For women who conceive via assisted reproductive technology (ART), there has been discussion and documentation on the toll that the process of fertility treatment has on patients' mental health [7, 8]. Additionally, the need to undergo ART treatment can compound the stressful nature of infertility on its own, thus predisposing patients to psychiatric sequalae [9]. However, previous literature has reported mixed findings on the risk for development of PPD, with most studies finding no difference, though these studies have been limited by small sample size [10, 11, 12]. The severity of PPD symptoms has also been investigated, with some data suggesting that patients who conceive with ART are not at an increased risk for a more severe presentation [13]. Studies on anxiety disorders in patients with conception via ART compared to spontaneous conception are largely focused on the experience of these disorders during pregnancy, but not in the postpartum period [14, 15]. Overall, literature on postpartum mood and anxiety disorders (PMADs) in the ART population is currently limited.

Outside of the context of ART, disparities in PPD have been documented across different races and ethnicities, with Black patients being more likely to report postpartum depressive symptoms than White patients [16]. The literature on Hispanic mothers is more mixed, but Asian‐American patients have been found to have lower rates of clinical PPD than non‐Hispanic White patients [16, 17, 18]. However, it is unknown whether these findings persist within a population of patients who conceived via ART.

The goal of this study was to utilize a large database of electronic health record (EHR) information to assess whether patients who conceive via ART are at a higher risk for PMADs. We also sought to determine whether there are any racial disparities for these conditions when conceiving with ART. We hypothesized that our large sample size would be able to overcome the limitations of prior studies and detect small but clinically significant differences in these subgroups, finding that patients who conceive via ART are at an increased risk for PMADs. We also hypothesized that Black women who conceive via ART have the greatest risk of developing PMADs while Asian women who conceive via ART conception have little to no risk of developing PMADs.

## 2 Materials and Methods

### 2.1 Database Description

We utilized the TriNetX research network to conduct this retrospective cohort study, accessing the database on December 11, 2025. We used the USA Collaborative Network within the TriNetX platform to form our patient cohorts. This network contains deidentified EHR data from over 100 million patients from 68 United States health care organizations in both the inpatient and outpatient settings. The data is deidentified per the Health Insurance Portability and Accountability Act (HIPAA) criteria—Section §164.514(a) of the HIPAA Privacy Rule. TriNetX has an exemption from the Western Institutional Review Board due to the data being deidentified, complying with HIPAA.

### 2.2 Study Population

**Figure 1:**
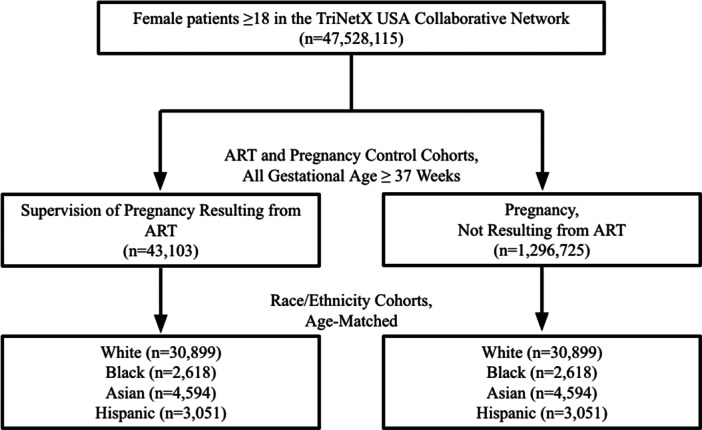
Flow diagram of study design. ART, assisted reproductive technology.

43,103 patients were included in the ART cohort, and 1,296,725 patients were included in the non‐ART cohort. Both cohorts comprised of only patients that reached 37 weeks gestational age using ICD‐10 codes Z3A.37‐39 and Z3A.4 (for a list of all codes used in the study, see Supporting Information Table S1). The ART cohort was defined using ICD‐10 code O09.81 (“supervision of pregnancy resulting from assisted reproductive technology”), representing patients who conceived from any form of ART, including intrauterine insemination (IUI) and in vitro fertilization (IVF). The non‐ART cohort was composed of patients with a history of pregnancy (ICD‐10 10 and Z33.1) and without a history of supervision of a pregnancy resulting from ART, defined by the aforementioned ICD‐10 code (Figure 1). Analysis was based on patients' first recorded ICD‐10 code in either category, so patients are not included multiple times in either group for subsequent pregnancies. Thus, we are unable to follow patients for subsequent pregnancies. Of note, ICD‐10 codes replaced ICD‐9 codes in October 2015, therefore the study population ultimately includes patients since the adoption of ICD‐10. Race and ethnicity‐specific cohorts were also created for both the ART and non‐ART populations.

### 2.3 Covariates and Outcomes

ART and non‐ART patients were matched for age at index event (history supervision of ART pregnancy and history of pregnancy respectively), race, ethnicity, history of major depressive disorder (MDD, ICD‐10 F33), history of generalized anxiety disorder (GAD, F41.1), and history of bipolar disorder (F31). For infertility‐matched analysis, we matched also for history of female infertility (N97), and for obstetric outcome‐matched analysis, we matched for multiple gestation (O30), pre‐eclampsia (O14), preterm delivery (O60.1), Cesarean delivery (CPT 1008991 or 1014218), and postpartum hemorrhage (O72) in the index pregnancy. Sub‐analyzes were displayed in addition to the fully matched analysis, which matched for all of the aforementioned variables, to elucidate if patient history of infertility of obstetric cofounders could be impacting an increased risk for postpartum mood disorders on their own (vs. undergoing the ART process).

We compared the prevalence of a depressive mood disorder composite consisting of PPD, MDD, or depressive episode, PPD on its own, and anxiety in the postpartum period (GAD) between the ART and non‐ART cohorts and between Black versus White, Asian versus White, and Hispanic versus non‐Hispanic patients in the ART cohort. The ICD‐10 codes used to define each outcome were F53.0 for PPD, F33 for MDD, F32 for depressive episode, and F41.1 for GAD. For the composite, a patient was classified as meeting the composite if they had any of the three diagnoses during the postpartum period, regardless of if they had one, two, or three of the diagnoses or the order in which multiple conditions were diagnosed. The postpartum time window for assessment of outcomes was 12 months. Therefore, outcomes had to occur within the 12 months following the inclusion ICD‐10 codes (ART/non‐ART pregnancy and gestational age of at least 37 weeks), and patients with outcomes prior to the index pregnancy were excluded. Only patients with gestational ages reaching 37 weeks were included because of an increased risk seen for PPD in patients who experience preterm delivery [19, 20]. No upper limit on gestational age at delivery was placed given that post‐term deliveries as these pregnancies have not been directly associated with PMAD, with the risk being largely explained by prenatal mental health diagnoses [21]. Secondary outcomes included a comparison by race and ethnicity between the ART and non‐ART cohorts, and comparisons across race and ethnicity cohorts of patients who all underwent ART (Black vs. White, Asian vs. White, and Hispanic vs. non‐Hispanic). A negative control outcome, injuries to the head (ICD‐10 S00‐S09), was also included to assess for detection bias.

### 2.4 Statistical Analysis

We compared the risk of the aforementioned outcomes between the cohorts using relative risk (RR) and 95% confidence intervals (CIs), and all analyzes mentioned were predetermined versus exploratory. Statistical analyzes were conducted in the TriNetX platform using R version 4.0.2, Survival package v3.2–3. Propensity score 1:1 matching used a greedy nearest neighbor approach with a caliper distance of 0.1 pooled standard deviation of the logit of the propensity score. Any characteristic with a standardized mean difference between cohorts lower than 0.1 is considered well matched [22]. Only propensity matching of 1:1 is completed in the TriNetX database, so other matching schemes, like 1:2, were unable to be completed. Balancing is displayed and evaluated within TriNetX, demonstrating significant differences between population characteristics (*p* < 0.001) prior to matching and non‐significant differences after matching (*p* < 0.05). *Z‐tests* were used to test the null hypothesis that the risk difference of each outcome was equal to 0. Confidence intervals for relative risks were based on Wald confidence limits [23]. The *p*‐values below 0.05 were considered statistically significant.

## 3 Results

1,192,013 patients were included in this study: 43,103 in the ART cohort and 1,296,725 in the non‐ART cohort. The mean [SD] age at index pregnancy of the ART cohort was 35.3 (5.0), and the mean [SD] age at index pregnancy of the non‐ART cohort was 29.0 [5.8]. Demographics of the ART cohort included patients who were 74.3% White, 6.8% Black or African American, 8.3% Asian, 0.3% American Indian/Alaskan Native, and 0.3% Native Hawaiian or Other Pacific Islander, 6.4% Hispanic, and 78.9% non‐Hispanic. In comparison, demographic characterization for the non‐ART cohort was significantly different than the ART cohort with patients who were 60.8% White, 17.3% Black or African American, 5.3% Asian, 0.7% American Indian/Alaskan Native, 1.1% Native Hawaiian or Other Pacific Islander, 19.3% Hispanic, and 62.3% non‐Hispanic. The baseline mental health diagnoses of the two cohorts were also significantly different prior to matching. Within the ART cohort, 3.4% of patients had MDD, 6.5% had GAD, and 1.0% had bipolar disorder. Conversely, 2.7% of patients had MDD, 4.5% had GAD, and 2.1% had bipolar disorder in the non‐ART cohort. Demographics for each cohort before and after matching are seen in Table 1, with the difference in matched‐for characteristics all reaching non‐significance after matching.

**Table 1:** Baseline characteristics of the study population before and after matching.

	Before matching			After matching		
	ART (*N* = 43,103)	Non‐ART (*N* = 1,296,725)	*p*‐value	ART (*N* = 42,827)	Non‐ART (*N* = 42,827)	*p*‐value
Age	39.3 ± 5.6	34.2 ± 6.4	< 0.001	NA	NA	NA
Age at index pregnancy	35.3 ± 5.0	29.0 ± 5.8	< 0.001	35.0 ± 4.8	35.0 ± 4.8	0.32
Race						
White	37,205 (74.3)	861,031 (60.8)	< 0.001	32,135 (75.0)	32,141 (75.0)	0.52
Black or African American	3417 (6.8)	244,689 (17.3)	< 0.001	2620 (6.1)	2604 (6.1)	0.73
Asian	4174 (8.3)	75,145 (5.3)	< 0.001	3411 (8.0)	3426 (8.0)	0.79
American Indian/Alaskan Native	135 (0.3)	9874 (0.7)	< 0.001	115 (0.3)	113 (0.3)	0.83
Native Hawaiian or Other Pacific Islander	159 (0.3)	15,217 (1.1)	< 0.001	148 (0.3)	148 (0.3)	0.52
Ethnicity						
Hispanic	3204 (6.4)	272,588 (19.3)	< 0.001	2983 (7.0)	2958 (7.0)	0.27
Not Hispanic	39,509 (78.9)	881,798 (62.3)	< 0.001	33,256 (77.7)	33,288 (77.7)	0.56
Pre‐existing Diagnosis						
Major Depressive Disorder	1466 (3.4)	35,110 (2.7)	< 0.001	1448 (3.4)	1461 (3.4)	0.69
Generalized Anxiety Disorder	2942 (6.8)	58,428 (4.5)	< 0.001	2915 (6.8)	2916 (6.8)	0.84
Bipolar Disorder	445 (1.0)	27,469 (2.1)	< 0.001	445 (1.0)	439 (1.0)	0.75

*Note:* Statistics presented as Mean ± SD, *N* (column %), NA – did not match for the variable.

Abbreviation: ART, Assisted reproductive technology.

After matching, there were 42,827 patients included in each cohort. Patients in the ART cohort were found to be at an increased risk for the depressive mood composite (5.9% vs 4.6%, RR 1.28 [95% CI (1.23,1.34)]), PPD (3.9% vs 3.4%, RR 1.13 [1.07,1.18]), and anxiety in the postpartum period (1.8% vs 1.2%, RR 1.56 [1.45,1.67]) (Table 2). With the addition of matching for both female infertility and obstetric cofounders, the ART cohort was still at an increased risk for all three outcomes: depressive mood disorder composite (5.9% vs 4.6%, RR 1.28 (1.20,1.36)), PPD on its own (3.9% vs 3.6%, RR 1.08 [1.01,1.16]), and anxiety in the postpartum period (1.8% vs 1.3%, RR 1.38 [1.24,1.54]). We conducted sub‐analyzes, matching based on history of female infertility and history of obstetric outcomes. For the infertility‐matched analysis, the ART cohort was at increased risk for the depressive mood disorder composite (5.8% vs 4.7%, RR 1.23 [1.16,1.31]) and anxiety in the postpartum period (1.8% vs 1.5%, RR 1.21 [1.10,1.34]). However, there was no difference in the risk of PPD on its own between the two cohorts (3.7% vs 3.7%, RR 1.01 [0.94,1.07]). There was not a significant difference between the ART and non‐ART cohorts for the negative control outcome (0.5% vs 0.6%, RR 0.84 [0.70,1.01], *p* = 0.07). For the obstetric outcome‐matched analysis, the ART cohort was again at an increased risk for the depressive mood disorder composite (5.8% vs 4.0%, RR 1.44 [1.35,1.53]), PPD on its own (3.7% vs 3.1%, RR 1.21 [1.13,1.30]), and anxiety in the postpartum period (1.8% vs 1.3%, RR 1.41 [1.27,1.57]).

**Table 2:** Risk of postpartum mood disorders in ART cohort versus non‐ART cohort following term delivery.

	ART	Non‐ART	RR	95% CI	*p*‐value
Overall, before matching, N (ART) = 43,103 N (Control) = 1,296,725					
Depressive mood disorder composite	2131 (5.9)	50,685 (4.6)	1.28	(1.23, 1.34)	< 0.001
PPD	1650 (3.9)	44,227 (3.4)	1.13	(1.07, 1.18)	< 0.001
Anxiety	740 (1.8)	14,669 (1.2)	1.56	(1.45, 1.67)	< 0.001
Matching by demographics, history of mental health conditions, female infertility, and obstetric cofounders, *n* = 41,775					
Depressive mood disorder composite	2076 (5.9)	1595 (4.6)	1.28	(1.20, 1.36)	< 0.001
PPD	1598 (3.9)	1474 (3.6)	1.08	(1.01, 1.16)	0.023
Anxiety	718 (1.8)	629 (1.6)	1.14	(1.03, 1.27)	0.015
Matching for female infertility, *n* = 48,760^a^					
Depressive mood disorder composite	2366 (5.8)	1887 (4.7)	1.23	(1.16, 1.31)	< 0.001
PPD	1785 (3.7)	1775 (3.7)	1.01	(0.94, 1.07)	0.86
Anxiety	819 (1.8)	675 (1.5)	1.21	(1.10, 1.34)	0.0002
Matching for history of obstetric cofounders, *n* = 49,729^a^^,^ b					
Depressive mood disorder composite	2414 (5.8)	1675 (4.0)	1.44	(1.35, 1.53)	< 0.0001
PPD	1837 (3.7)	1516 (3.1)	1.21	(1.13, 1.30)	< 0.0001
Anxiety	843 (1.8)	595 (1.3)	1.41	(1.27, 1.57)	< 0.0001

*Note:* N (% of cohort), RR, relative risk, 95% CI, 95% confidence interval.

Abbreviations: ART, assisted reproductive technology; PPD, postpartum depression.

^a^ Also matched for age, race, ethnicity, and history of anxiety, depression, bipolar disorder.

^b^ Obstetric co‐founders consist of multiple gestation, pre‐eclampsia, preterm delivery, Cesarean delivery, and postpartum hemorrhage.

We then compared the prevalence of these outcomes between patients who did and did not undergo ART within the same racial and ethnic groups (Table 3). After matching for age at index pregnancy and for race/ethnicity, the number of patients in each cohort was: 30,899 White patients, 2618 Black patients, 4594 Asian patients, and 3051 Hispanic patients. Within the White cohort, there was an increased risk for all three measures in patients who underwent ART, with the prevalence of the depressive mood composite being 5.6% in the ART cohort versus 3.7% in the non‐ART cohort (RR 1.49 [1.38, 1.61]). For PPD on its own, the prevalence was 3.1% in the ART cohort compared to 2.8% in the non‐ART cohort (1.12, [1.03, 1.23]), and for anxiety in the postpartum period, the prevalence was 1.6% in the ART cohort versus 1.2% in the non‐ART cohort (RR 1.39 [1.21, 1.60]). These findings persisted in the Asian cohort for the depressive mood disorder compositive (3.0% vs 1.4%, RR 2.16 [1.60, 2.92]), PPD (1.8% vs 1.2%, RR 1.48 [1.04, 2.08]), and anxiety in the postpartum period (0.6% vs 0.3%, RR 2.00 [1.06, 3.80]). For the Hispanic cohort, ART patients were at an increased risk for the depressive mood disorder composite (4.2% vs 3.0%, RR 1.38 [1.05, 1.83]) and anxiety in the postpartum period (1.0% vs 0.4%, RR 2.59 [1.33, 5.02]), but not PPD on its own. For the Black cohort, ART patients were only at an increased risk for the depressive mood disorder composite (4.6% vs 3.3%, RR 1.41 [1.05, 1.88]) but not PPD on its own or anxiety in the postpartum period.

**Table 3:** Risk of postpartum mood disorders in ART cohort versus non‐ART cohort by race/ethnicity.

	ART	Non‐ART	RR	95% CI	*p*‐value
White, *n* = 30,899^a^					
Depressive mood disorder composite	1472 (5.6)	971 (3.7)	1.49	(1.38, 1.61)	< 0.001
PPD	965 (3.1)	858 (2.8)	1.12	(1.03, 1.23)	0.011
Anxiety	475 (1.6)	341 (1.2)	1.39	(1.21, 1.60)	< 0.001
Black, *n* = 2618^a^					
Depressive mood disorder composite	107 (4.6)	76 (3.3)	1.41	(1.05, 1.88)	0.020
PPD	78 (3.0)	58 (2.2)	1.35	(0.96, 1.88)	0.081
Anxiety	21 (0.8)	13 (0.5)	1.62	(0.81, 3.22)	0.17
Asian, *n* = 4594^a^					
Depressive mood disorder composite	131 (3.0)	61 (1.4)	2.16	(1.60, 2.92)	< 0.001
PPD	83 (1.8)	56 (1.2)	1.48	(1.06, 2.08)	0.021
Anxiety	28 (0.6)	14 (0.3)	2.00	(1.06, 3.80)	0.030
Hispanic, *n* = 3051^a^					
Depressive mood disorder composite	113 (4.2)	83 (3.0)	1.38	(1.05, 1.83)	0.022
PPD	84 (2.8)	82 (2.7)	1.03	(0.76, 1.39)	0.86
Anxiety	31 (1.0)	12 (0.4)	2.59	(1.33, 5.02)	0.004

*Note:* N (% of cohort), RR, relative risk, 95% CI, 95% confidence interval.

Abbreviations: ART, assisted reproductive technology; GAD, generalized anxiety disorder; MDD, major depressive disorder; PPD, postpartum depression.

^a^ Matching re‐run for race/ethnicity cohorts.

Finally, we assessed for any racial or ethnic differences in the prevalence of these outcomes, primarily looking at patients who underwent ART. Asian patients were significantly less likely than White patients to develop the depressive mood disorder composite (3.0% vs 5.4%, RR 0.54 [0.44, 0.67]), PPD (1.8% vs 2.7%, RR 0.67 [0.51, 0.88]) and anxiety in the postpartum period (0.6% vs 1.5%, RR 0.42 [0.27, 0.66]). There was no significant difference between the prevalence of any of the outcomes between White and Black and Hispanic and non‐Hispanic patients who underwent ART. We also ran a comparison between the non‐ART cohorts and found Black (3.0% vs 3.5%, RR 0.87 [0.83, 0.90]), Hispanic (2.2% vs 3.7%, RR 0.59 [0.57, 0.61]), and Asian (1.6% vs 2.7%, RR 0.60 [0.55, 0.65]) patients to all be less likely to be diagnosed with PPD than their comparison group (White or non‐Hispanic).

## 4 Discussion

Our study compared the risk of developing a depressive mood disorder composite, PPD, and anxiety in the postpartum period in patients who conceived via ART and naturally following a term delivery. Before and after matching for age, race/ethnicity, and history of MDD, GAD, and bipolar disorder, we demonstrated an increased risk for the development of the depressive mood disorder composite, PPD on its own, and anxiety in the postpartum period in patients who conceive via ART. These findings persisted when we matched patients for both a history of negative obstetric outcomes commonly associated with ART, and PPD, as well as a history of female infertility. Despite these findings being statistically significant, the magnitude of the difference is small and arguably not clinically significant. The absolute difference in prevalence between our matched cohorts was only 1.3% (for the depressive mood disorder composite) but under a percent for PPD and anxiety in the postpartum period. We additionally examined if these findings persisted across racial and ethnic groups, finding that all groups (White, Black, Asian, and Hispanic) who conceived with ART were more likely to develop a disorder within the depressive mood disorder composite and anxiety in their postpartum period than their natural conception counterparts. However, Black and Hispanic patients who conceived via ART were not at an increased risk for developing PPD on its own compared to the non‐ART cohort. Finally, within the ART cohort, Asian patients were found be less likely to develop the depressive mood disorder composite, PPD, and anxiety in the postpartum period. No differences were documented between White and Black and non‐Hispanic and Hispanic patients.

With a study population including over 40,000 patients who conceived via ART, our study is the largest (to our knowledge) to consider PMAD diagnoses in patients who conceived via ART. Reassuringly for both providers and patients, the overall risk of developing PPD is slightly higher but overall similar to the general population. This adds clarity to the literature, as prior studies demonstrated mixed results regarding if ART increases the risk for PPD [10, 11, 12].

By including sub‐analyzes matching for either history of female infertility or history of obstetric outcomes commonly associated with ART, we hoped to elucidate whether either of these factors were the cause for the increased risk of PMADs in our study. Matching for infertility on its own, without the obstetric outcomes, yielded no significant difference in PPD between the two cohorts, but findings persisted for the depressive mood disorder composite and anxiety in the postpartum period. However, we are unable to elucidate varying degrees of infertility in our analysis, meaning a difference could still be seen in patients who are struggling to conceive for a longer period. By also only including patients who had a gestational age of 37 weeks or greater, we wanted to try and control for the increased risk of preterm delivery seen with ART conception. Thus, our study has not elucidated a specific factor associated with ART that may be putting patients at this slightly increased risk for PMADs. Potentially, the stressors of the ART process, psychological, financial, and physical, especially in combination with infertility, could be examined in the future. Nevertheless, these findings call for a need for increased mental health services for patients undergoing ART, both during treatment and in the postpartum period.

Our race and ethnicity sub‐analysis findings are dissimilar to previous studies. We hypothesized that Black patients would be more likely than White patients to experience PMADs based on literature in related areas of study, which was not found in our data [16, 17]. However, these disorders have been found to be significantly more underdiagnosed in Black patients compared to White patients, even within a cohort of patients who all screened positive for postpartum mood and anxiety symptoms, so the difference may be understated due to disparities in diagnosis [24]. Another possible reason for underdiagnosis of PMADs in Black patients is a reluctance for these patients to communicate mental health symptoms with their providers based on numerous factors like a lack of provider diversity, stigma around mental illness, previous negative healthcare experiences, or even a fear of providers contacting child protective services due to their symptoms [25, 26]. Previous literature comparing Hispanic and non‐Hispanic patients has shown a reduced risk of PPD in Hispanic patients compared to White patients, which was seen in our analysis comparing non‐ART groups [17]. However, this comparison has not been conducted within a group of patients who all conceived via ART, so it is plausible that the disparity seen in the general population does not occur in the context of ART. Hispanic patients have also been found to report more depressive symptoms at 2 weeks postpartum [16]. We did not find a similar increase of depressive orders in the postpartum period in Hispanic ART patients compared to their non‐Hispanic counterparts, but we also looked at a 12‐month period postpartum and not within a few weeks after delivery.

For Asian patients, ART conception was associated with an increased risk of all outcomes assessed, in line with the rest of the race/ethnicity cohorts. However, within the ART cohort, these patients were significantly less likely to have postpartum depression and anxiety diagnoses compared to their White counterparts. These findings are in line with our initial hypothesis due to several previous studies reporting lower rates of PPD in Asian mothers [17, 18]. It is possible that this difference is related to the observation that depression and mental health conditions are more stigmatized in Asian culture [27, 28]. As a result, it has been found that Asian patients are less likely than their White counterparts to report and seek out mental health services for depressive symptoms [29]. Therefore, it is difficult to discern if Asian patients are actually at a lower risk for PPD because the difference may be from patients being less likely to share mental health symptoms with their providers, leading to underdiagnosis. Additionally, the limitation of a broad, heterogenous racial classification like “Asian” should be considered in the context of our results, as mental health beliefs differ across different Asian cultures [30]. For this population, the focus should be on providing culturally competent care and recognizing that these patients may be less likely to openly discuss mental health concerns [31].

Our study has several notable strengths, such as the sample size generated by using a large database, yielding a cohort of over 40,000 patients who have conceived via ART. However, it is not without limitations. One limitation of this study is the reliance on ICD‐10 coding for analysis. PPD and anxiety in the postpartum period appear to be underreported compared to the estimated prevalence of these conditions in the United States. To try and address this limitation, we included the depressive mood disorder composite in case these conditions were coded differently across institutions. Additionally, there is a chance PMADs are reported at a higher rate in the ART cohort given the higher‐risk nature of these pregnancies, potentially leading to closer postpartum monitoring [32]. We tried to reduce biases pertaining to the inherent demographic differences between the cohorts by matching for age, race, ethnicity, and history of several mental health conditions, but we were unable to control for socioeconomic status, social support, and geographic location. Other covariates that we were unable to assess, but could impact our findings, include parity, gestational number, sexual orientation, gender identity, history of pregnancy loss, ART type (e.g., IVF vs. IUI, etc), non‐ART fertility treatments, ART care that was not coded within our chosen cohort definition, length of infertility, male versus female infertility, psychotropic medications during pregnancy, potential diagnostic biases, and healthcare utilization in the postpartum period. An additional limitation of our study is its retrospective design as an observational study. Therefore, we cannot attribute causation to the reported findings.

In summary, our study demonstrates that, while conception via ART is associated with a statistically increased risk for the development of PMADs, this difference is small and arguably not clinically significant. Nevertheless, clinicians should bring awareness to PMADs in the prenatal period to destigmatize these conditions, and practices should try to offer mental health services to patients suffering from infertility. Increased vigilance is critical in screening high‐risk patient populations for mood disorders at postpartum visits, perhaps considering earlier screening. White, Black, Asian, and Hispanic patients who conceive via ART are all at increased risk, suggesting a benefit to standardized screening protocols regardless of patient demographics. For Asian patients, cultural values may contribute to the disparity in postpartum mood disorders observed in this study. Thus, clinicians and health systems should seek to provide care in a culturally competent manner to provide the best care to all patients who conceive via ART.

## Author Contributions

Marisa R. Imbroane contributed to conceptualization, investigation, methodology, data curation, formal analysis, and writing (original draft, review, and editing). Hanna Kim contributed to conceptualization, methodology, formal analysis, and writing (review and editing). Elliott G. Richards contributed to conceptualization, methodology, formal analysis, and writing (review and editing).

## Disclosure

All authors have read and approved the final version of the article. Marisa R. Imbroane had full access to all of the data in this study and takes complete responsibility for the integrity of the data and the accuracy of the data analysis.

## Conflicts of Interest

The authors declare no conflicts of interest.

## Ethics Statement

Ethical approval was not required, as the TriNetX database has an exemption from the Western Institutional Review Board given that all data are properly anonymized to comply with HIPPAA.

## Transparency Statement

The lead author Marisa R. Imbroane affirms that this article is an honest, accurate, and transparent account of the study being reported; that no important aspects of the study have been omitted; and that any discrepancies from the study as planned (and, if relevant, registered) have been explained.

## Supporting information


**Table S1:** ICD‐10/CPT Code List.

## Data Availability

The data that support the findings of this study are available from TriNetX. Restrictions apply to the availability of these data, which were used under license for this study. Data are available from the author(s) with the permission of TriNetX.
