# Supplementary material for: Risk of Mood and Anxiety Disorders in the Postpartum Period Following Assisted Reproduction: A Retrospective Cohort Study
Source: Health Sci Rep. 2026 Apr 2;9(4):e72169. doi: 10.1002/hsr2.72169 (PMC13052237; doi:10.1002/hsr2.72169)
Supplement: Supplementary file 1 — Table S1: ICD‐10/CPT Code List. [file HSR2-9-e72169-s001.docx]

Supp Table 1: ICD-10/CPT Code List

| Diagnosis/Outcome/Characteristic | Code(s) |
| --- | --- |
| **Cohort Creation/Inclusion Criteria** |  |
| 37 weeks gestational age | ICD-10 Z3A.37-39, Z3A.4 |
| Supervision of pregnancy resulting from assisted reproductive technology | ICD-10 O09.81 |
| History of pregnancy | ICD-10 10, Z33.1 |
| **Characteristics for Matching** |  |
| Major depressive disorder | ICD-10 F33 |
| Generalized anxiety disorder | ICD-10 F41.1 |
| Bipolar disorder | ICD-10 F31 |
| Female infertility | ICD-10 N97 |
| Multiple gestation | ICD-10 O30 |
| Pre-eclampsia | ICD-10 O14 |
| Preterm delivery | ICD-10 O60.1 |
| Cesarean delivery | CPT 1008991 or 1014218 |
| Postpartum hemorrhage | ICD-10 O72 |
| **Outcomes** |  |
| Depressive mood disorder composite | ICD-10 F53.0 or F33 or F32 |
| Postpartum depression | ICD-10 F53.0 |
| **Negative Control Outcome** |  |
| Injuries to the head | ICD-10 S00-S09 |
